# Is it ever morally permissible to select for deafness in one’s child?

**DOI:** 10.1007/s11019-019-09922-6

**Published:** 2019-09-21

**Authors:** Jacqueline Mae Wallis

**Affiliations:** Department of Philosophy, University of Bristol, Bristol, BS6 6JL UK

**Keywords:** Deafness, Deaf, PGD, Disability, Elizabeth Barnes, Value-neutral model

## Abstract

As reproductive genetic technologies advance, families have more options to choose what sort of child they want to have. Using preimplantation genetic diagnosis (PGD), for example, allows parents to evaluate several existing embryos before selecting which to implant via in vitro fertilization (IVF). One of the traits PGD can identify is genetic deafness, and hearing embryos are now preferentially selected around the globe using this method. Importantly, some Deaf families desire a deaf child, and PGD–IVF is also an option for them. Selection for genetic deafness, however, encounters widespread disapproval in the hearing community, including mainstream philosophy and bioethics. In this paper I apply Elizabeth Barnes’ value-neutral model of disability as mere-difference to the case of selecting for deafness. I draw on evidence from Deaf Studies and Disability Studies to build an understanding of deafness, the Deaf community, and the circumstances relevant to reproductive choices that may obtain for some Deaf families. Selection for deafness, with deafness understood as mere-difference and valued for its cultural identity, need not necessitate impermissible moral harms. I thus advocate that it is sometimes morally permissible to select for deafness in one’s child.

Reproductive genetic technologies continue to advance, providing families with increasing options to choose what sort of child they want to have. Preimplantation genetic diagnosis (PGD), for example, allows parents to evaluate several existing embryos and select which to implant via in vitro fertilization (IVF) (Sermon et al. 2004). Current technologies are limited and carry various burdens, some of which are quite serious. Nonetheless, our options will plausibly continue to increase in breadth, efficacy, and safety; all this renders the ethical questions about how these technologies should be used even more pressing.^1^

Saliently, genetic loci for hereditary deafness have been identified and can be used in PGD (Camporesi 2010). In most cases, embryos possessing congenital markers for deafness are not selected.^2^ This is plausibly because, in the hearing community, deafness is often understood to be a disability that as such should not be chosen for one’s child using a method such as PGD. For example, prominent philosopher and bioethicist Julian Savulescu (2002, p. 771) writes that “[m]any would see deliberately creating deaf babies as the most perverse manifestation of creating designer babies”, and that “[m]any people believe that doctors should not help couples to have a deaf child.”

Conversely, in the Deaf community some parents prefer to have a deaf child.^3^ A well-known case of a Deaf couple selecting for deafness (though not via PGD) is profiled in Mundy (2002).^4^ Tucker (1998) reports that Deaf families using genetic counseling services at Gallaudet University, a liberal arts university for the Deaf in the United States, often want deaf rather than hearing babies.^5^

In one of the few investigations stratifying parental preferences with respect to disability more generally, Baruch et al. (2008, p. 1055) report that, in the United States, “[t]hree percent of IVF–PGD clinics report having provided PGD to couples who seek to use PGD to select an embryo for the presence of a disability.” Note that this percentage represents couples who *sought* to use PGD in this way, not the percentage of cases in which selection for disability actually occurred; nonetheless, this report suggests that the preference to select for disability is neither widespread nor trivial. To illustrate how difficult disability can be to define, especially in the context of reproductive genetic technologies, Dance (2017) cites the case of deafness: “one person’s disability can be another person’s culture or community—for example, in the case of close-knit communities like the deaf. Some deaf parents even request PGD so that they can ensure their children will be born deaf, and thus take part in their culture and lifestyle.”

Many Deaf people report that they view Deafness more as a unique culture and identity than as an impairment (Grodin and Lane 1997). The historic oppression of Deaf people, which includes institutionalized efforts by the hearing majority to control their reproduction (Biesold 1999), in addition to the views of some scholars in Deaf Studies (Boudreault et al. 2010) and Disabilities Studies (Wasserman and Asch 2012) about the importance of this issue, raise the question of whether commonplace assumptions about the impermissibility of selecting for deafness are legitimate.

In this paper I first examine several common objections to selecting for deafness (“The source(s) of moral harm”).^6^ Each of the proposed moral harms, I attempt to show, assumes or otherwise involves an understanding of deafness as a “bad-difference”, as the sort of condition which makes an overall negative difference in one’s life. “Initial arguments in favor” reviews initial arguments in favor of selecting for deafness, and suggests that, when they fail to challenge the bad-difference view, they are inadequate. “The value-neutral model of disability” presents and briefly summarizes Elizabeth Barnes’ (2009, 2014, 2016) value-neutral model of disability, which holds physical disability to be “mere-difference”, a trait which simply *makes a difference* and is *neutral* with respect to overall well-being. “The case of selecting for deafness” applies Barnes’ model to the case of selecting for deafness.

It is in “The case of selecting for deafness” that I make my argument that yes, it is sometimes morally permissible to select for deafness in one’s child. Supporting evidence begins accumulating, however, as I respond to the arguments in “The source(s) of moral harm”, “Initial arguments in favor” and “The value-neutral model of disability”. Briefly, I argue that deafness, insofar as it is a disability, is mere-difference and thus neutral with respect to overall well-being. Being deaf does not necessarily cause substantial, overall harm in a person’s life. In fact, Deaf families can possess sufficient lived experience and general knowledge to evaluate what harms, if any, could accrue to their choice to select for deafness in a future child. In making a selection choice between several embryos, some of which carry genes for genetic deafness and some which do not, parents choosing on the basis of expected well-being may thus be morally permitted to select an embryo with genetic deafness.^7^

## The source(s) of moral harm

In its most basic sense, ‘deafness’ designates the state of being deaf, i.e., lacking the sense of hearing. ‘Hearing loss’ and ‘hearing impairment’ generally refer to medical conditions, and people with different amounts of hearing can identify as Deaf. Some people who become deaf later in life identify as “hearing people with a disability” (Lane 2005). Important for the trajectory of my argument is the inclusion of a social dimension: deafness is precisely the feature identified and used by the deaf community.^8^ “The source(s) of moral harm” describes some of the most common objections to selecting for genetic deafness in the context of a technology such as PGD.

To begin, consider this representative claim from Trevor Johnston (2005, p. 429): “insofar as deafness is a disability, it is to be avoided, if possible.” Implicit in this simple argument is that disability is, in itself, bad. This assumption is consistent with the model of disability that Elizabeth Barnes calls the bad-difference view of disability. Under bad-difference views, disability “is something inherently bad for you” (Barnes 2016, p. 54). The bad-difference view of disability is often associated with a medicalized model of disability. Dominant in medical and healthcare settings (in addition to popular understandings), the medical model attempts to give a naturalistic account of disability but often connotes the physical differences we term “disabilities” as somehow suboptimal, dysfunctional, or based in deficit.^9^ At first glance, it seems that the “badness” of disability does the moral work to prohibit selecting for deafness. I will argue, however, that assuming deafness is necessarily bad in order to justify that selecting for deafness is bad is *begging the question*.

The question we actually need to get at is: where precisely is the moral harm, if any, in selecting for deafness? Three loose categories can capture the most prevalent objections in the literature; I will call these “Harming the child”, “Restricting future life plans”, and “Introducing a harmful state of affairs”. I treat each in turn, outlining the objection and giving examples.

Actually, there is a fourth common category of objections in the literature, those which concern responsibilities and virtues. These arguments usually cite specific parental responsibilities and/or virtues in domains of civic and family life. Both McDougall (2007) and Fahmy (2011) address parental virtues in conjunction with the case of selecting for deafness. Due to length constraints I here omit consideration of objections from responsibilities and virtues; these objections either seem not to contain the assumption of bad-difference about deafness I wish to address or contain the assumption via one of the domains I survey in “Harming the child”, “Restricting future life plans”, and/or “Introducing a harmful state of affairs”. A fuller account of the moral permissibility of selecting for deafness, however, will benefit from addressing parental virtues.

### Harming the child

One objection to selecting for deafness claims that, in choosing deafness for one’s child, one harms the child in an important way. Selecting a deaf embryo is a harm because the future child will lack one of the central human sense modalities. This harm constitutes sufficient reason to deem selecting for deafness morally impermissible, as parents should not intentionally harm their children if they have reasonable alternatives (e.g., the other nondeaf embryos). Setting aside the fact that the argument from direct harm also begs the question, it has at least one simple dismissal: “[a]s many authors have acknowledged,” summarizes Melissa Seymour Fahmy (2011, p. 129), “the child in question is not injured or made worse off by the selection or resulting birth, given that the only alternative for the child would be not to exist at all.” This sort of reply, which we will see again in “Restricting future life plans”, is usually attributed to Derek Parfit and is known as the non-identity problem.^10^

Julian Savulescu and Guy Kahane (2009, p. 276) argue that, in considering direct harm to the child, it would be morally impermissible to select a child only if its life “would be so bad it is not worth living.” And clearly, they state, “[t]he lives of the vast majority of disabled people are not merely worth living but good.”^11^ In the case of deafness, the testimony of the Deaf community,^12^ the existence of the Deaf Pride movement,^13^ much scholarship,^14^ and one might argue common sense, all support that the life of a deaf person is indeed worth living. So, it cannot be the case that, in selecting for deafness, one harms the child in this substantial sense. But, Savulescu and Kahane seem to have set what Joseph Stramondo (2017, p. 482) calls “an incredibly low bar” here, as “the non-identity problem would find any choice acceptable so long as the resulting life is not so burdened by suffering that it is not worth living.”

Another way to respond to the harming-the-child objection is to show that it might be possible that it is actually a good thing to be deaf. Parents who select for deafness are not doing serious harm to their child because being deaf actually accrues benefits and goods, not harm. Rachel Cooper (2007) explores this in “Can it be a good thing to be deaf?”. Cooper gives reasons why being deaf might be a good thing, categorized under language, qualia, and culture; I will briefly mention these and a few others.

First, signed languages are now recognized as full natural languages (Sacks 1991, p. 90). Any language can have advantages and disadvantages with respect to other languages, Cooper asserts, which suggests signed languages have some advantages over spoken languages. One example could be the “unique, additional powers [of Sign] of a spatial and cinematic sort—at once a most complex and yet transparent expression and transformation of thought” (Sacks 1991, p. 90). There are likely some unique advantages of signed languages which only accrue to those for whom Sign is their first and primary language.

In terms of sense perception, deaf people can have enhanced visual and vibrational qualia experiences. A deaf child can be referred to (quite descriptively, note Grodin and Lane) as a “visual child” (Grodin and Lane 1997). Deaf people are “visual people” with specially developed visual abilities.^15^ Further, “if the primary language is Sign,” writes Sacks (1991, p. 111), summarizing findings in neurology, “there will be, additionally, an enhancement of many sorts of visual-cognitive ability, all going along with a shift from right hemisphere to left hemisphere dominance.” Considering enhanced vibrational qualia, Sacks (1991, p. 8n) writes: “those with the profoundest deafness […] may be highly sensitive to vibrations of all kinds. This sensitivity to vibration can become a sort of accessory sense: thus Lucy K., although profoundly deaf, can immediately judge a chord as a ‘fifth’ by placing a hand on the piano and can interpret voices on highly amplified telephones; in both cases what she seems to perceive are vibrations, not sounds.”

Cooper notes that having more qualia is not always better than having fewer; overload and confusion are possible. I came across a great illustration of this in a recent New York Times obituary for Kitty O’Neill, “Stuntwoman and Speed Racer” (Sandomir 2018). The columnist writes, “[b]eing deaf, [O’Neill] often said, helped deepen her concentration, whether she was racing a dragster or leaping off buildings.” O’Neill still holds the land-speed record for women; in December 1976 she rode a “three-wheeled rocket-powered vehicle” across the desert in Oregon, peaking at 618 m.p.h. and attaining “an average speed of 512.7 m.p.h., shattering the land-speed record for women by about 200 m.p.h.” O’Neill’s testimony suggests that lacking sound qualia enhanced her ability to perform stunts and pursue her daring career.

In terms of culture, giving a single evaluation of the importance (and limitations) of Deaf culture would be impossible. Certainly, many Deaf people report valuing Deaf culture very highly (Grodin and Lane 1997). In *Seeing Voices* Oliver Sacks (1991, p. 114n) reports another cultural-linguistic advantage of deafness: “[t]here is no one universal signed language. And yet there may be universals *in* signed languages, which help to make it possible for their users to understand one another far more quickly than users of unrelated spoken languages could understand each other.” Signers thus have an advantage over speakers of spoken languages when it comes to traveling and encountering people who use languages different from their own.

Cooper (2007, pp. 579, 580) comes to a nuanced conclusion “that whether it is a good or bad thing to be deaf is hard to determine. Plausibly, being deaf may be a bad thing for some deaf people but not for others.” Cooper acknowledges that Deaf scholars will likely be capable of proposing a more comprehensive list of “deaf goods” that could be used to evaluate the question further within a philosophical framework. One such example exploring deaf goods is Bauman and Murray (2014). If it is indeed the case that being deaf is not always a bad thing (all things considered), then we might be more justified in believing that selecting for deafness does not necessarily guarantee the kind of substantial, overall life harm that would make such a selection morally impermissible. This is not to say that the decision to select an embryo with genetic deafness is equally *risky* as selecting genetically nondeaf embryos.^16^

### Restricting future life plans

A second kind of objection to the moral permissibility of selecting for deafness is that parents unjustly restrict their child’s potential life plans. Fahmy (2011) attributes the origin of this argument to Dena Davis and Joel Feinberg. In choosing deafness for one’s child, the objection goes, the child’s right to an open future is unnecessarily violated. Silvia Camporesi (2010) makes an argument of this sort in “Choosing Deafness with Preimplantation Genetic Diagnosis: An Ethical Way to Carry On a Cultural Bloodline?”. Indeed, Camporesi writes, “I framed the issue in terms of justice toward the future children, as I argued that choosing deafness with PGD is unjust toward them because it is a broad limitation to a ‘reasonable array of different life plans’” (ibid, p. 93). Sara Goering, in her Stanford Encyclopedia of Philosophy article “Eugenics” (2014, pp. 14, 15), summarizes a similar argument from Dena Davis: “deliberately trying to conceive a child who will have relatively limited options—limited, in [Davis’s] view, not just due to enduring discrimination and social constraints, but also to bodily deficits—is morally impermissible.” Some of the life plan restrictions a deaf child might face include limited communication with peers who cannot Sign, reduced ability to pursue some careers (e.g., music), dependence on accommodations for some aspects of education, cultural events, navigation, safety, etc.

Those who make this argument can concede that a deaf life is worth living, and that deaf people can have good, fulfilling lives. However, the argument turns on the notion that being deaf will unfairly confine that child to a narrower choice of familial interactions, educational opportunities, career paths, social lives, etc. Deaf advocates can reply that being deaf opens future life plans that being hearing cannot, and that families who seek to choose deafness for their child do not view this as a limitation (Camporesi 2010). Reasonably, one might also argue that the kind of constraint that being deaf might impose on one’s life choices cannot be weighed against the other kinds of constraints that parents knowingly and unknowingly impose on any children they choose to have.

Interestingly, Nicholas Agar (2004), who maintains a life-plan-limitation view, also holds, as cited by Goering (2014, p. 14), that “in some circumstances, lacking a capacity like hearing will only lead to a small difference in relative freedom, and if so, then we might not have a ‘general requirement to replace genetic arrangements linked with deafness’ (105).” I thus suggest that the life plan restrictions of being deaf, at least for some children, may not be substantial enough to necessarily entail impermissible moral harm. Indeed, “[m]ost people with disabilities find that their conditions do not limit their ability to enjoy life, though most people without disabilities do not believe them” (Bagenstos and Schlanger 2007, p. 797).

More abstractly, results in cognitive science do not indicate that increased opportunities correlate linearly with increased well-being.^17^ Excessive multiplicity of choice can cause anxiety or be overwhelming. Robert Sparrow (2002, p. 11) raises a related point in “Better off Deaf” when he observes, “[i]n a society which fetishises individual choice and opportunity, it may seem obvious that these are goods.”^18^ Perhaps more options only increase our happiness to a point, and after that point, increasing options are neutral with regards to happiness. Without solid evidence, however, we ought not insist that the reduced magnitude of future openness for a deaf child could be grounds to make selection for deafness necessarily impermissible. Moreover, we might develop a system whereby breadth and worth of opportunities are distinguished and weighed separately. I expect that sufficient and worthwhile potential life plans exist for a deaf person who is the child of Deaf adults in a Deaf family, learns Sign as their first language, is raised in the Deaf community, and lives in a larger society which is accepting and accommodating to deaf people and Deaf culture.^19^

But what about Deaf parents raising a hearing child in the same way? This objection is considered by Camporesi:

I can concede […] the point that the deaf culture may have its compensations that hearing people cannot fully experience, but the fundamental point here is that deaf parents do not need to choose to exclude their children from the hearing world in order to include them in theirs (however, imperfectly include them, as parents may counterargue), because both worlds and languages are open to their children: both the hearing and the not hearing worlds, both the verbal and the nonverbal languages. (2010, p. 90)

The point Camporesi raises here, that Deaf parents could include a hearing child in Deaf culture (by teaching them Sign, acquainting them with Deaf people, getting them involved in Deaf community events etc.) is frequently asserted. There is good counterevidence, however, from children of Deaf adults (CODAs) that this is indeed challenging and “imperfect” (Mand et al. 2009). Plausibly, Deaf parents already are simultaneously aware of this “bicultural” option, to raise a hearing child with aspects Deaf culture, and also of the diverse experiences of CODAs in their own communities. The fact that some parents *still* prefer a deaf child is telling. Perhaps there is an asymmetry introduced when a hearing child is included in Deaf culture; namely, the parents cannot fully engage their child with respect to the child’s *hearing culture*. For example, when the child makes hearing friends who cannot Sign, the Deaf parents face communication barriers with those friends and probably their parents, too. The hearing child will have many experiences which the Deaf parents might have difficulty sharing or helping them with.

Regardless of problems with the restricting-future-life-plans objection, Fahmy points out that the argument can be set aside in the same manner as harming-the-child:

The decision that purportedly curtails the child’s right to an open future is simultaneously the decision which allows the child to exist and to have any future whatsoever […] The decision to select for deafness thus […] cannot be said to deprive the resulting child of opportunities he or she would have otherwise been able to pursue. (Fahmy 2011, p. 130)

This is the Parfit non-identity problem again.

### Introducing a harmful state of affairs

Even once we can dismiss the notions that selecting deafness harms a child or restricts their options, a third objection remains. This objection does not focus on the child per se, but on the general circumstances, or state of affairs, brought about by the parental choice to select for deafness. By selecting deafness, the argument goes, the family introduces into the world a harmful state of affairs, and this harm is importantly avoidable (should a nondeaf embryo be alternatively selectable). There will be suffering and/or limited opportunity in the deaf child’s future world which could have been avoided in the selection of a different child. This formulation avoids the Parfit non-identity problem, which we could use to dismiss the first two kinds of objections. However, the harmful-state-of-affairs objection faces its own issues.

Firstly, David Wasserman and Adrienne Asch (2012, p. 30) hold that even if parents select a better overall state of affairs (by not selecting deafness, for example), “we do not think they should be seeking to improve the world at large in deciding whether to gestate and raise a child.” Reproductive decisions, in their view, should not require parents to bring about a particular state of affairs. So, one way to address the worry of introducing a potentially harmful state of affairs is to deny that parents should be primarily concerned with influencing “the world at large”. In other words, it cannot be the case that selecting for deafness is always impermissible, at least on the grounds that some harmful state of affairs obtains as a result, because choosing a particular embryo is not the kind of decision which requires parents to improve the broader state of their society or world.

Secondly, one might argue that allowing families to select for deafness places an undue burden on society, since society will be responsible for providing many of the accommodations, such as specialized education and interpreting services, that the child will grow to need. I think this concern is largely unwarranted. It is not at all clear that any prospective parents could make the balancing calculations required to evaluate the future impacts of their children. Characterizing this worry in another way Fahmy (2011, p. 132) asks, “[h]ow much anticipated suffering and/or limited opportunity is enough to suggest a moral obligation to avoid this harm by substituting one potential child for another, or by forgoing procreation altogether?” This becomes a threshold problem. Determining what is morally permissible depends on setting a threshold at some particular level. A state of affairs approach is therefore troubling if one is unwilling to choose a threshold for what sorts of potential harms constitute a state of affairs that it is morally impermissible not to avoid.^20^

Lastly, it is also useful to note that some use the state of affairs approach to condemn selecting *against disabilities* at all. For example, Barnes (2016, p. 153) argues that, while selecting against disability might not be morally impermissible, it is at least morally blameworthy insofar as it can promote societal stigmas about disability and “communicate ableism”. In a similar vein, “[d]ifference and diversity,” write Grodin and Lane (1997, p. 248), “not only have evolutionary significance but, we would argue, are a major part of what gives life its richness and meaning.” Indeed, they conclude that “how we treat this problem will say a great deal about what kind of society we are and the kind of society in which we wish to live” (ibid). The idea here is that disability actually improves the overall state of affairs in the world.

The use of state-of-affairs reasoning to criticize selection *against* disability highlights how the introducing a harmful state-of-affairs objection to selecting *for* deafness errs by assuming that selecting a deaf child will necessarily introduce a “harmful state of affairs”. This is precisely the intuition that we have been starting to challenge throughout “The source(s) of moral harm”. Even if it were true, however, that selecting deafness introduced harm in the world, it is somewhat trivial; every reproductive decision will plausibly introduce *some* harm into the world. Every child’s life will include some limited opportunities and suffering, determined by climate, politics, socioeconomic status, biology, etc. Granted, these limitations are differentially modulated by parental decisions, but they interact in complex ways regardless of disability status. All the preceding considerations suggest that evaluating impact on the overall state of affairs will not establish definitively that selecting deafness is morally impermissible.

To conclude “The source(s) of moral harm”, I emphasize that all the supposed moral harms of selecting for deafness in some way rely on assumptions about the inherent “badness” of being deaf (or its consequences). What happens if we question the bad-difference view? I return to this explicit question in “The value-neutral model of disability”. Meanwhile, in “Initial arguments in favor”, I present some initial arguments in favor of selecting for deafness, which run contrary to the objections presented here in “The source(s) of moral harm”.

## Initial arguments in favor

For some, the preceding objections to selecting for deafness simply do not obtain: “If physicians are willing to perform PGD to select for hearing children,” state Wasserman and Asch (2012, p. 30), “they should be willing to perform PGD to select for deaf children.” As I have cited previously, physicians do indeed select for hearing children. Let us see how the argument in favor of selection for deafness might begin.

Sparrow (2002, p. 14) poses the following question: “could parents ever have good grounds for believing that their child would be ‘better off deaf’—where ‘better off’ is determined with reference to the worth of the range of opportunities they will have to lead a good human life? Yes.” Sparrow believes there are two sorts of reasons to believe this: cultural identification and opportunities. The former—that Deaf parents might believe their way of life to be culturally more valuable—Sparrow believes to be less plausible but is nonetheless reluctant to dismiss. The latter, however, he thinks quite compelling: Deaf parents “might prefer a deaf child, not because they believe that the ways of life promoted in Deaf culture are more valuable than those promoted outside of it, but simply because they are *capable of being much better parents* to a child who belongs wholeheartedly to their own (Deaf) culture” (ibid, p. 15, emphasis mine). He then goes on to give a good account of why a deaf child parented by Deaf adults might benefit to a much greater extent than a hearing child would. In conclusion, Sparrow holds:

If we acknowledge the reasonableness of a cultural understanding of Deafness and an identification with Deaf culture, and if we allow the use of genetic technologies to parents wishing to have children of a certain sort, then we have no legitimate grounds to deny Deaf parents the right to use these technologies in order to have deaf babies, if they wish to do so. (2002, p. 16)

While I generally agree with Sparrow here, one can argue that this view neglects to consider appropriately the biological nature of disability, or the impacts of hearing impairment that we may wish to account for. Matti Häyry takes a stance similar to Sparrow’s:

Those who believe that we should always produce “the best children we can” are likely to argue, against my conclusions, that deafness is a disability, and that we should never deliberately bring disabled individuals into existence. My question to them is, why not? All human beings live the best life they can, and if life is a good thing, then why deprive some potential individuals of that opportunity because of their personal qualities? (2004, p. 511)

Both Sparrow (2002) and Häyry (2004) exhibit views of deafness that are opposed to the bad-difference view of disability. But as Johnston (2005, p. 434) notes, “[t]he disadvantages an individual may experience due to a disability are not purely the result of the social construction of that disability.” Deaf people, though they have meaningful and good lives, “can still experience a sense of limitation, disadvantage, and disability because of their biology, which is additional to socially constructed stigma and disabling prejudice that deaf advocates and social theorists have documented” (ibid, p. 428). For example, a deaf person may feel limited by the fact that they cannot listen to birdsong or symphonies the way hearing people do. So, it seems we require an account of disability, and of deafness, that moves beyond the bad-difference view, but maintains an embodied element. That is indeed what I propose the value-neutral model can do.

## The value-neutral model of disability

Many of the arguments in “The source(s) of moral harm” against selecting for deafness hinge on the impact of deafness on well-being. When a negative, harm, and deficit-based (i.e., bad-difference) view of disability is used to understand deafness, the answer to whether it is morally permissible to select for a deaf child seems to be a simple no.^21^ Selecting deafness seems to inflict impermissible harm on one’s future child. However, this is again begging the question, and we should wish to avoid doing this. What precisely do we mean by “disability” and what are its implications? We cannot *assume* that disability is necessarily an overall “bad” and then justify our rejection of selecting for deafness simply because we label it a disability. Instead we need to examine, and perhaps change, our concept of disability. We can then discern the relevance of disability, in this case deafness, to a future child’s well-being.

### Erik Parens on a “binocular” view of disability

Erik Parens (2017) examines the concept of disability in “Choosing Flourishing: Toward a More ‘Binocular’ Way of Thinking about Disability”. He notes that Disability Studies scholars and some philosophers have proposed analyses similar to his (ibid, p. 143). While Parens cites Elizabeth Barnes, he does not mention her value-neutral model of disability, perhaps to the detriment of his own argument.

Parens proposes what he calls a more “binocular” view of disability. His choice of term is slightly unfortunate in the way that it centers a metaphor of vision, but the invitation to expand our conception of disability is a promising one. Parens’ argument turns on the notion that when parents choose features of their potential child, they are not choosing in an “all else being equal” environment (ibid, p. 141). This “all else being equal” often plays a role in arguments *against* choosing disability, because the premises assume that making the choice between particular features can be made independently of all other considerations. In Parens’ view, the choice takes place within a framework that renders a condition typically considered to be a disability into something else. “When people speak of choosing deafness or short stature,” writes Parens, “they are not choosing those traits because they take them to be disabilities. They are choosing those traits *because they take them to be enhancements*, where by ‘enhancement’ I mean an intervention that will facilitate a child’s or a family’s flourishing, as they understand it” (ibid, p. 44, emphasis mine). So, according to Parens, a couple that selects for deafness in their child is, in a sense, selecting not for disability, but instead for flourishing. Here again we see the assumption that disability, as a kind of bad-difference, must be opposed to well-being; this is because (for Parens) selecting for disability and selecting for flourishing are mutually exclusive. Parens’ evaluation, though, does seem consistent with some Deaf people’s desires during genetic counseling. I. King Jordan (1991), scholar and first Deaf president of Gallaudet University, reports that Deaf couples, even if neutral about whether or not their child is deaf, often want a child without other traits, such as motor impairment, which are perceived as undesirable disabilities in the Deaf community.

Unfortunately, the way Parens’ account erases disability in the case of selection is not ideal. His conception of selecting disability does not actually challenge the bad-difference view; it seems instead to assume that some disabilities (such as deafness or dwarfism) can be flourishing-promoting “enhancements”. A different theory of disability may provide more traction on the question of selecting for genetic deafness. The situation identified by Parens, however, that parents who choose an attribute such as deafness are in a sense choosing something positive (flourishing) for their child, is something we should keep in mind. Perhaps the contribution here is simply a reminder to acknowledge that, when it comes to their potential future children, disabled parents can be thoughtful, dedicated, and invested choosers. Our task is now to describe an account of disability which neither assumes the bad-difference view nor fails to adequately capture what a disability like deafness is (and thus, how it impacts on well-being and selection decisions).

### Elizabeth Barnes on minority bodies: disability as value-neutral mere-difference

In “Disability, Minority, and Difference” Elizabeth Barnes (2009) outlines the value-neutral model of disability that she extensively develops in her book *The Minority Body: A Theory of Disability* (2016). In both cases, she gives reasons why she addresses the model to physical disabilities only.^22^ In the remainder of this section I will give a brief account of Barnes’ model.^23^

The central move Barnes (2016, p. 55) makes is to characterize disability as difference, or more relevantly, as *mere* difference: “having a disability makes you physically non-standard, but it doesn’t (by itself or automatically) make you worse off.” Barnes’ proposal counters the bad-difference view. Disability is the kind of feature, so the bad-difference view usually goes, that makes an overall negative difference in one’s life; that is, to have a disability is to have a suboptimal body, to have a harmful overall deficit in one’s biology. Further, “[a]ccording to bad-difference views of disability, not only is having a disability bad for you, having a disability would still be bad for you even if society was fully accommodating of disabled people” (Barnes 2014, p. 89).

Barnes cites the testimony of disabled people themselves and extensive literature on this topic—more widely acknowledged since the rise of Disability Studies—to show that disabled people do not typically have an overall negative understanding of their health or well-being.^24^ Contrary to what the bad-difference view suggests about their disabilities, most people with disabilities do not describe themselves as suboptimal, deficient, dysfunctional, etc. but rather as healthy, whole, functional, etc. Although this does not establish that disabled people are or are not any of these things (there are multiple, interacting issues when it comes to self-assessments of well-being), Barnes reminds readers that “[t]he intuitions of the (privileged) majority don’t have a particularly good track record as reliable guides to how we should think about the minority, especially when the minority is a victim of stigma and prejudice” (Barnes 2016, p. 156).

Barnes (2016) suggests that we can better understand this apparent “discrepancy” by distinguishing between what she calls “local bads” and “global bads”. Precisely: “Φ is *locally bad* for x iff Φ has a negative effect on x’s well-being with respect to some feature *F* or some time *t*. Φ is *globally bad* for x iff Φ has a negative effect on x’s overall well-being” (Barnes 2016, pp. 80, 81). No one will deny that disabilities can cause local harms; there are many ways, both due to biology and to the social world, in which everyday living as a disabled person can be difficult. For example, a deaf person may experience a local bad when an important town hall meeting fails to provide sign language interpreters. However, the presence of local bads does not necessitate that disability will cause *global* harm.

The distinction between local and global harms expands into one between narrow and overall well-being (and was hinted at by Parens, treated above).^25^ There are many goods that can arise from disability, and disabled people can have meaningful and happy lives, like everyone else.^26^ “[J]ust because disability takes away a freedom (creates a limitation),” writes Barnes (2009, p. 347), “doesn’t allow us to conclude that it makes a person worse off. The same feature that takes away a freedom (creates a limitation) could create other freedoms (prevent other limitations) elsewhere.” Furthermore, Barnes argues that “[w]hether Φ is good/bad for x is not merely a function of whether Φ is itself good or bad simpliciter with respect to well-being. Whether Φ is good/bad for x is also—and in large part—a matter of what else (both intrinsic and extrinsic) it is combined with” (Barnes 2016, p. 86). So, it cannot be the case that disability is necessarily a bad-difference *on the whole*. Instead, disability should be characterized as a mere-difference, or value-neutral with respect to overall well-being. On this view deafness would not be characterized as a deficit or a suboptimal condition, but rather as a particular sort of “minority body” (Barnes 2016, p. 6).

The biggest (immediate) challenges for the value-neutral model concern causing and removing disability.^27^ Barnes uses principles from reproductive ethics to argue that causing disability, even if considered a mere-difference for the potential bearer, would not be permissible if the harms caused were serious enough. Thus “it’s impermissible to cause disability for the very same reasons,” Barnes (2009, p. 349) writes, “that it’s impermissible to allow disability. It’s impermissible because it’s impermissible to *cause* a person (particularly one’s own child) serious harm—even if that harm stands a good chance of being outweighed by other benefits.”

Deafness, however, is a condition that does not cause serious overall harm and suffering; therefore, I argue, in an open, accepting, and accommodating society, parents might not be obligated to choose a hearing embryo over a deaf one.^28^ In the case of deafness the potential *local* harms to the child do not seem serious enough (deafness does not cause constant pain, for example) to warrant an injunction against selecting for it. In terms of *global* harms, the testimony and scholarship about Deafness suggests that deafness itself is not such a harm. As Parens suggests, reasonable parents can desire a deaf child over a hearing one from a positive mindset about their child’s potential to flourish in their family and community.

Barnes admits that she desires a successful value-neutral account to condemn negative selection *against* disability but concedes that her account cannot maintain this. The most her account can do is hold it morally *blameworthy* to select against disability. Motivations to select against disability seem grounded in the bad-difference view, a view Barnes (2009, p. 350) calls “a profound misunderstanding.” In her view selecting against disability is thus “to import disparaging views about disability (that it is something sub-optimal, rather than just something different)—and *that* is blameworthy” (Barnes 2009, p. 351).

One of the strongest objections to a mere-difference view comes from Jeff McMahan (2005). Setting aside how one considers a single disability, McMahan says, it is clear that multiple disabilities aggregate into an overall bad-difference. It is not the case that adding several “neutral” disabilities results in a merely different kind of life for the bearer of those disabilities. Barnes (2009, p. 352) responds in part by saying that “[i]f individual disabilities involve local harms, then those harms will naturally add up—having lots of disabilities will likely lead to lots of harm. But the crucial point here is just that this doesn’t license the conclusion, *for any particular disability*, that it will make a person worse off on the whole.” This also goes back to Barnes’ argument that whether a disability is good/bad *for* someone depends on what it is combined with. Deafness, as a single disability, seems different from the deafness experienced by a deaf-blind person, for example. Because deafness, when combined with another disability (such as motor impairment), might seem especially harmful, does not mean that deafness, when considered individually, is defined by that level of harm.

Another response to McMahan’s objection is that the harm accruing to multiple disabilities is an emergent phenomenon, i.e., a new effect accruing to a set of causes and not merely a sum of the individual effects of those causes. Singly, a disability can be neutral with respect to overall well-being, but perhaps, summed together, multiple disabilities can interact to produce substantial, emergent harm. One of the mechanisms by which this could occur is the compounding reduction of capabilities that a person might use to address local harms. Emergent properties are more than the sum of their parts, so in this way we can account for the seriousness of multiple disabilities without designating individual disabilities as bad-differences. I do not think that McMahan’s objection from multiple disabilities should require us to reject Barnes’ analysis of particular disabilities, especially in the narrow case being considered in this paper.

Barnes (2009, p. 352) concludes with the following words: “[m]any things make life harder; but they can also enhance and enrich it. Disability is just one of many such features—the sorts of things that create difficulty and hardship, but which make the world a more interesting and vibrant place in the process.” With her value-neutral model of disability as mere-difference, understanding disabled people as people with minority bodies, we have good reasons for both mainstream philosophy and applied ethics to make a shift in their understanding of disability. Now, equipped with this model, we can further develop the case of selecting for deafness and see how the value-neutral account helps to support the moral permissibility of this reproductive choice.

## The case of selecting for deafness

Savulescu and Kahane, scholars who argue strongly in favor of technologies like PGD, write: “[w]hat determines whether there are moral reasons for or against selecting a child with a congenital condition such as deafness is factual information about the expected well-being of such a child, when compared to other possible children” (2009, p. 290). The context for this statement is what they call a strong conclusion: “parents have significant reasons to select the most advantaged children” (ibid, p. 289). This conclusion, however, pivots crucially on what we understand to be an “advantage” for a child; the value-neutral model of disability will help us more reasonably weigh the aspects of deafness relevant to overall well-being. As Cooper (2007) argues, there are plausibly for some deaf people benefits to outweigh the challenges of being deaf. How does a value-neutral model help us establish the moral permissibility of selecting for deafness? Let us continue.

First, a value-neutral model of deafness better aligns with many testimonies of deaf people, as I briefly demonstrate in what follows.^29^ Importantly, summarize Vehmas and Shakespeare (2014, p. 43), “Deaf advocates consider Deafness to be a minority cultural experience based on shared language, not an impairment.” This evaluation is also expressed, for example, by Grodin and Lane (1997, p. 234): “[b]eing DEAF is highly valued in DEAF culture. DEAF people who espouse those cultural values are glad they are DEAF, and they reject the suggestion that they have an impairment or a disability.” They add revealingly that the American Sign Language sign for disability “does not include being DEAF” (ibid, p. 234).

Because many Deaf advocates define deafness without using a deficit or dysfunction model, and because their evaluation arises from the lived experiences of Deaf people, the next step might be to deny entirely, as evident in the quotation above, the “disability” label.^30^ In the bad-difference view of disability, “disability” is a stigmatized label that as such becomes less useful to actual disabled people: “[i]n the case of Deaf culture, Deaf people seek to separate themselves from the societal concept of disability altogether thereby removing the stigmatizing label” (Jones 2002, p. 57). The mere-difference view, with a value-neutral model, fits better and could allow the hearing and Deaf communities alike to understand deafness as a disability without assuming disparaging views about disability and the lives of disabled people. For example, Rebecca Atkinson, who identifies as a “partially deaf person”, writes in a post for the BBC’s disability blog *Ouch!* that:

[…] any claim that “I’m not disabled, I’m Deaf” should be made with care, not least because in saying so you appear to take the stance that disability is such an abhorrent state that you wish to disassociate yourself from it. To me, though, the term “disability” should not be synonymous with low status, failure and undesirability, but instead signify pride and solidarity. Disability should be part of the spectrum of human life, upon which we all, deaf included, stand. (Atkinson 2008)

Atkinson’s testimony fits with Barnes’ model.

Barnes’ value-neutral model is consistent with much scholarship on disability; Vehmas and Shakespeare, for example, state that “deafness may be a disadvantage, but not a disadvantage *on balance”* (2014, p. 44, emphasis mine). They argue that the disability movement helped show the distinction “between impairment or disability as a form of harm—which we would not contest—and between disability as a harmed life” (ibid, p. 45). As I read it, a “form of harm” is a type which can locally cause harm (and is presumably experienced by disabled and nondisabled people), whereas a “harmed life” is a life determined by, and most explanatorily described by, harm. A “form of harm” versus a “harmed life” dovetails nicely with Barnes’ distinction between local harm and global harm. Barnes relies on the convincing testimony of many disabled people to create her model.^31^ Barnes also uses Miranda Fricker’s (2007) notion of epistemic injustice to present why the nondisabled majority ought to listen to the disabled consensus about the kind of difference that disability, as such, is.

In addition to better capturing a relevant Deaf understanding of deafness, the value-neutral model provides other advantages. We have already seen how the objections to selecting for deafness are largely inadequate, but the value-neutral model further challenges them. I argue that choosing deafness for one’s child is not harming the child—in a sense *significant enough* to warrant moral impermissibility—because deafness should be understood holistically as a mere-difference, not a bad-difference. Next, contra the restricts-future-life-plans responses, we can recognize that choosing deafness, as a mere-difference, is neutral with respect to future overall well-being; thus, it is not the case that a life with deafness causes a *harmful* reduction of possibilities. In fact, under the value-neutral model, we might more readily identify all the new opportunities and possibilities that open to a particular child should she be born deaf. Important considerations about deafness and Deaf culture, combined with the mere-difference view, will also help show how choosing deafness does not, qua deafness, introduce a “harmful state of affairs”; quite the opposite, actually, as we might be more open to understanding the perspective of the Deaf parents who believe they are, in Parens’ words, “choosing flourishing” for their child. Understanding disability itself as mere-difference rather than bad-difference allows us to more justly weigh the advantages and disadvantages of deafness for a particular potential child.

To put it simply, we should reject the notion that a nondeaf life is clearly better than a deaf life. Instead, we ought to recognize that some families may have good, morally-grounded reasons for selecting genetic deafness for a future child. Stramondo considers a case of using PGD to select for disability (achondroplasia) and similarly authorizes parents:

We can endorse their decision to use PGD to deliberately have a child with achondroplasia in as far as we can be reasonably sure that these parents have a handle on what material, epistemic, and emotional resources will be needed to accommodate the child’s modes of functioning and whether or not these resources will be available in the context in which they will raise the child. (2017, p. 488)

## Conclusion

In “Can It Be a Good Thing to Be Deaf?” Rachel Cooper (2007) explains why we are probably ill-equipped to find this answer the “easy” way. The “easy” way, in Cooper’s estimation, would be either to ask deaf people themselves or to appeal to some understanding of the “natural”. The former is limited because no single person can possess (and thereby compare) the complete, lifelong experiences of two inherently different (e.g., deaf and hearing) phenomenal lives. The latter—appeal to the “natural”—fails for the following reason: “[t]here is no necessary link between an organism functioning properly in a biological sense and being in a good state. As such, asking whether deafness is a biological dysfunction will not help determine whether it is a bad thing to be deaf,” suggesting the independence of biological functioning and human flourishing (ibid, p. 570). Cooper highlights that, though this debate hinges on broader questions about the nature of human flourishing, we should be able to examine each of the advantages and disadvantages of being deaf in order to build a more accurate overall understanding.

In this paper I applied Elizabeth Barnes’s value-neutral model of disability as a mere-difference to the case of selection for genetic deafness. Weighing advantages and disadvantages of being deaf using this model—and doing so in consultation with lines of thought from Deaf Studies and Disability Studies—helps address the epistemic injustice of doubting disability testimony. Barnes (2016, p. 143) argues “that both causing disability and removing disability are complex issues, and that the mere-difference view doesn’t entail a universal response to either.” In other words, the specifics of each case (of selection, for example) will inform the moral evaluation.

My aim here was to suggest that, in the case of genetic deafness and PGD, it may sometimes be morally permissible for parents to select deafness for their future child. I recognize this places significant responsibility on parents to appropriately consider the kind of life their future child will have. Deaf parents are duly capable. It is my hope that the reader now better understands how and why some parents might select for deafness. And I further advocate that this selection, contrary to widespread hearing intuition, be considered morally permissible.

The author of this paper is hearing.

## Footnotes

1 For an overview of reproductive ethics see Kukla and Wayne (2018), and for a disability view see Shakespeare (2016) and (2011). In “Better beings?”, a recent News Feature in Nature Biotechnology, Amber Dance (2017) investigates some of the ethical questions raised by new reproductive technologies.

2 For example, Xiong et al. (2015) and Yazdi et al. (2018) recently report selection against deafness.

3 I loosely follow the practice to use ‘Deaf’ with an uppercase ‘D’ to refer to the culture and identity of Deafness, and to the people who identify with that culture, and otherwise to use ‘deaf’ with a lowercase ‘d’ to refer to the biological condition of being deaf, or to refer to people who may or may not identify as Deaf.

4 See Savulescu (2002) for early commentary on this case.

5 See also Middleton et al. (1998) and Stern et al. (2002).

6 The question of selecting for deafness via PGD is separate from the questions concerning the morality of using reproductive technologies in general. One who believes that we should not use these technologies at all must needs attend to the present argument via syllogism, i.e., *if* PGD–IVF is accepted and allowed, *then* selecting for deafness is/is not morally permissible. However, because such technologies are already widely in use, I here address the specific case of the moral permissibility of selecting for deafness. See Stramondo (2017) for an ethical analysis of PGD in the disability context.

7 NB: In the United States, there are no specific laws governing the selection of genetically “anomalous” embryos. In the United Kingdom, there is a stricter mandate not to select an embryo which would significantly increase the chance that the child will develop a serious disability, serious illness, or other serious mental condition (American Society for Reproductive Medicine Ethics Committee 2017). For a brief summary of global legal approaches to the regulation of PGD see Camporesi (2010).

8 See Lane (2005) for a more comprehensive and nuanced summary of the English term ‘deaf’ and its relation to the Deaf-World (in the United States particularly), “citizens whose primary language is American Sign Language (ASL) and who identify as members of that minority culture” (p. 291).

9 The philosopher of biology Tim Lewens, when describing biological functions, writes: “The biologist who wishes to understand changes in the frequencies of traits in a population has no need to decide which should count as malfunctioning, and which should count as functioning: it is enough to construct a model that can explain trait fluctuations” (Lewens 2007, p. 550). I will note that there is an exception for health and disease, however deafness is clearly not a disease (at least it is not necessarily a disease). So, per Lewens, the call to label the disability of deafness a *biological malfunction* is arguably unwarranted.

10 See Parfit (1984) and Buchanan et al. (2000). For a contrary view, that coming into existence is itself always a harm, see Benatar (2007).

11 See, for empirical support, Albrecht and Devlieger (1999) and Frederick and Loewenstein (1999).

12 Variously evident, for example, in Bauman (2008), Mundy (2002) and Sacks (1991).

13 See, for example, Kannapell (1980) and Ladd (2003).

14 See, for example, Bauman (2008) and Grodin and Lane (1997).

15 Here I do not mean to insinuate that the unique goods or experiences of deafness *alone* justify selecting a deaf embryo over a hearing one. Instead, my intent is to suggest that when evaluating selection of a disability such as deafness, we ought not exclude consideration of the potential (and often unique) gains that accrue to some disabilities in some contexts.

16 I do not further consider the issue of risk, but see Barnes (2014, pp. 96–103) for a discussion in the disability context.

17 See Schwartz (2004) for the definitive book describing the phenomenon whereby “more” choice becomes psychologically harmful. See Schwartz and Ward (2004) for book chapter review of the topic.

18 See Sparrow (2002) for a good argument for why we cannot be obligated to maximize our children’s future opportunities, but rather ought to ensure they have worthwhile ones. The objection here uses a negative approach, that moral harm arises from limiting opportunities (and not from failing to maximize opportunities). Stramondo (2017) makes a similar argument, arguing that parents are not required to choose the “best” future children. Instead, they should be allowed to select based on their idea of the good life, even if this includes disability. Stramondo’s account revolves around the notions of opportunity *ranges* and reasonable accommodation (as opposed to *greatest* opportunity and normal species functioning).

19 See Mundy (2002) for a compelling and heartfelt illustration.

20 Another angle on the state of affairs objection might take us to procreation and the environment in general. In debates about procreation from the viewpoint of climate ethics, for example, many argue that having children is analogous to overconsumption—because each child will have its own large carbon footprint (Young 2001). Incidentally, the difference between a disabled and nondisabled child in terms of magnitude of environmental impact is nowhere near the difference between a child and no child at all.

21 Recall, for example, Johnston (2005): “insofar as deafness is a disability, it is to be avoided, if possible.” (p. 429).

22 The two main reasons, Barnes (2016, p. 2) explains, are [1] simplicity and [2] epistemology of testimony. Note that she does not hold that the model cannot apply to other disabilities but rather that more work is needed to expand it to a wider range of disabilities.

23 Barnes uses a social constructivist, solidarity-based understanding of disability but finds the social model—i.e., the collection of views associated with the distinction between [A] individual impairments and [B] the social, prejudicial causes of disabling situations—unsatisfactory (Barnes 2016, p. 25). For more on the social model see Shakespeare and Watson (2002).

24 Barnes cites Bagenstos and Schlanger (2007) for an overview of the relevant literature. “A massive body of research,” those authors conclude, “has demonstrated that people who acquire a range of disabilities typically do not experience much or any permanent reduction in the enjoyment of life” (ibid, p. 763). Another review cited by Barnes is Frederick and Loewenstein (1999). See also Albrecht and Devlieger (1999). Of course, “enjoyment of life” may not be one’s preferred criterion for evaluating well-being; the research is nonetheless suggestive that many nondisabled intuitions about the lives of disabled people are incorrect and subject to stigma.

25 Some harms are non-experiential and thus do not impact well-being per se. I do not consider non-experiential harms here but thank Havi Carel for bringing up this point.

26 For example, explore the phenomenon known as “Deaf Gain” which “refers to the unique cognitive, creative, and cultural gains manifested through deaf ways of being in the world” (Bauman and Murray (Eds.) 2014, xv). “So-called baby-sign programs for hearing infants and their parents are one of the most widely known and lucrative examples of Deaf Gain” (ibid, p. 146).

27 These are addressed in *The Minority Body*, Chapter 5: Causing Disability (Barnes 2016).

28 See “The case of selecting for deafness” for my further development of this argument.

29 Sharanjit Uppal (2006), to provide a broad example, reports a large study of subjective well-being of Canadians with disabilities. The study contained approximately 24,000 people, of whom about 30% self-identified as having a “hearing disability”; among these, the results in answer to the question “Overall how would you say things are these days?” were: (20.9%) very happy; (65.9%) pretty happy; (13.2%) not too happy. Subjective well-being for people with a hearing disability (which I assume includes both deaf and Deaf people) were similar to those in the entire disability population in the study and, Uppal notes, citing a similar survey carried out in a similar year, to the general population in Canada. For testimonies from the Deaf Studies perspective see, for example, Kannapell (1980), Ladd (2003), Bauman (2008) and Bauman and Murray (2014).

30 Disability Studies scholar Megan Jones (2002, p. 51) summarizes and gives ample citations for “views on deafness as a culture [that] have recently emerged that consider deafness as a trait, not as a disability.”

31 The disability population is diverse, and no one view can represent every disabled person’s experience. However, Barnes (2016) draws on extensive literature to emphasize the point that “disabled people do not rate themselves as being in general less happy or satisfied with life than do non-disabled people” (p. 93n12). The review she cites at this point is Frederick and Loewenstein (1999). “And there is more than just these first-person reports of well-being,” Barnes (2016, p. 119) notes later, “[t]here is art, there is theater, there is dance, there is film, there are parades—all celebrating disability as a valuable form of diversity.” I take “a valuable form of diversity” as additional support for the mere-difference (rather than bad-difference) view.

## References

[CR1] Agar Nicholas (2004). Liberal eugenics: In defence of human enhancement.

[CR2] Albrecht Gary L, Devlieger Patrick J (1999). The disability paradox: High quality of life against all odds. Social Science and Medicine.

[CR3] American Society for Reproductive Medicine Ethics Committee (2017). Transferring embryos with genetic anomalies detected in preimplantation testing: an Ethics Committee Opinion. Fertility and Sterility.

[CR4] Atkinson, Rebecca. 2008. Is deafness a disability? Ouch! It’s a disability thing. April 2. http://www.bbc.co.uk/ouch/features/is_deafness_a_disability.shtml. Accessed Jan 3 2019.

[CR5] Bagenstos Samuel R, Schlanger Margo (2007). Hedonic damages, hedonic adaptation, and disability. Vanderbilt Law Review.

[CR6] Barnes Elizabeth (2009). Disability, minority, and difference. Journal of Applied Philosophy.

[CR7] Barnes Elizabeth (2014). Valuing disability, causing disability. Ethics.

[CR8] Barnes Elizabeth (2016). The minority body: A theory of disability.

[CR9] Baruch Susannah, Kaufman David, Hudson Kathy L (2008). Genetic testing of embryos: Practices and perspectives of US in vitro fertilization clinics. Fertility and Sterility.

[CR10] Bauman H-Dirksen L (2008). Open your eyes: Deaf studies talking.

[CR11] Bauman H-Dirksen L, Murray Joseph J (2014). Deaf gain: Raising the stakes for human diversity.

[CR12] Benatar David (2007). Better never to have been: The harm of coming into existence.

[CR13] Biesold Horst (1999). Crying hands: Eugenics and deaf people in Nazi Germany.

[CR14] Boudreault Patrick, Baldwin Erin E, Fox Michelle, Dutton Loriel, Tullis LeeElle, Linden Joyce, Kobayashi Yoko (2010). Deaf adults’ reasons for genetic testing depend on cultural affiliation: Results from a prospective, longitudinal genetic counseling and testing study. The Journal of Deaf Studies and Deaf Education.

[CR15] Buchanan Allen, Brock Dan W, Daniels Norman, Wikler Daniel (2000). From chance to choice: Genetics and justice.

[CR16] Camporesi Silvia (2010). Choosing deafness with preimplantation genetic diagnosis: An ethical way to carry on a cultural bloodline?. Cambridge Quarterly of Healthcare Ethics.

[CR17] Cooper Rachel (2007). Can It be a good thing to be deaf?. Journal of Medicine and Philosophy.

[CR18] Dance Amber (2017). Better beings?. Nature Biotechnology.

[CR19] Fahmy Melissa Seymour (2011). On the supposed moral harm of selecting for deafness. Bioethics.

[CR20] Frederick Shane, Loewenstein George, Kahneman D, Diener E, Schwarz N (1999). 16 hedonic adaptation. Well-Being. The foundations of Hedonic Psychology.

[CR59] Fricker Miranda (2007). Epistemic injustice: Power and the ethics of knowing.

[CR21] Goering, Sara. 2014. “Eugenics.” *The* Stanford Encyclopedia of Philosophy (Fall 2014 Edition). Edited by Edward N. Zalta. July 2. https://plato.stanford.edu/archives/fall2014/entries/eugenics/. Accessed Nov 20 2018.

[CR22] Grodin Michael, Lane Harlan (1997). Ethical issues in cochlear implant surgery: An exploration into disease, disability, and the best interests of the child. Kennedy Institute of Ethics Journal.

[CR23] Häyry Matti (2004). There is a difference between selecting a deaf embryo and deafening a hearing child. Journal of Medical Ethics.

[CR24] Johnston Trevor (2005). In one’s own image: Ethics and the reproduction of deafness. The Journal of Deaf Studies and Deaf Education.

[CR25] Jones Megan A (2002). Deafness as culture: A psychosocial perspective. Disability Studies Quarterly.

[CR26] Jordan I King (1991). Ethical issues in the genetic study of deafness. Annals of the New York Academy of Sciences.

[CR27] Kannapell Barbara, Baker C, Battison R (1980). Personal awareness and advocacy in the deaf community. Sign language and the deaf community: Essays in honor of William C. Stokoe.

[CR28] Kukla, Rebecca, and Katherine Wayne. 2018. Pregnancy, birth, and medicine. In *The Stanford Encyclopedia of Philosophy,* ed. Edward N. Zalta. March 21. https://plato.stanford.edu/archives/spr2018/entries/ethics-pregnancy/. Accessed 1 Feb 2019.

[CR29] Ladd Paddy (2003). Understanding deaf culture: In search of deafhood.

[CR30] Lane Harlan (2005). Ethnicity, ethics, and the deaf-world. The Journal of Deaf Studies and Deaf Education.

[CR31] Lewens Tim, Matthen M, Stephens C (2007). Functions. Philosophy of biology: Handbook of the philosophy of science.

[CR32] Mand C, Duncan RE, Gillam L, Collins V, Delatycki MB (2009). Genetic selection for deafness: The views of hearing children of deaf adults. Journal of Medical Ethics.

[CR33] McDougall Rosalind (2007). Parental virtue: A new way of thinking about the morality of reproductive actions. Bioethics.

[CR34] McMahan Jeff (2005). Causing disabled people to exist and causing people to be disabled. Ethics.

[CR35] Middleton Anna, Hewison Jenny, Mueller Robert F (1998). Attitudes of deaf adults toward genetic testing for hereditary deafness. The American Journal of Human Genetics.

[CR36] Mundy, Liza. 2002. A world of their own. *The Washington Post*, March 31.

[CR37] Parens Erik (2017). Choosing flourishing: Toward a more ‘binocular’ way of thinking about disability. Kennedy Institute of Ethics Journal (Project MUSE).

[CR38] Parfit Derek (1984). Reasons and persons.

[CR39] Sacks Oliver (1991). Seeing voices.

[CR40] Sandomir, Richard. 2018. Kitty O’Neil, Stuntwoman and Speed Racer, Is Dead at 72. *New York Times*, November 6. https://nyti.ms/2yWaSr8.

[CR41] Savulescu Julian (2002). Deaf lesbians, ‘designer disability’, and the future of medicine. BMJ.

[CR42] Savulescu Julian, Kahane Guy (2009). The moral obligation to create children with the best chance of the best life. Bioethics.

[CR43] Schwartz Barry (2004). The paradox of choice: Why more is less.

[CR44] Schwartz Barry, Ward Andrew, Linley PA, Joseph S (2004). Doing better but feeling worse: The paradox of choice. Positive psychology in practice.

[CR45] Sermon Karen, Van Steirteghem André, Liebaers Inge (2004). Preimplantation genetic diagnosis. The Lancet.

[CR46] Shakespeare Tom (2011). Choices, reasons and feelings: Prenatal diagnosis as disability dilemma. ALTER European Journal of Disability Research.

[CR47] Shakespeare Tom (2016). Just what is the disability perspective on disability?. Hastings Center Report.

[CR48] Shakespeare Tom, Watson Nicholas (2002). The social model of disability: An outdated ideology?. Research in Social Science and Disability.

[CR49] Sparrow Robert (2002). Better off deaf. Res Publica.

[CR50] Stern SJ, Arnos Kathleen S, Murrelle L, Oelrich Welch K, Nance Walter E, Pandya Arti (2002). Attitudes of deaf and hard of hearing subjects towards genetic testing and prenatal diagnosis of hearing loss. Journal of Medical Genetics.

[CR51] Stramondo Joseph (2017). Disabled by design: Justifying and limiting parental authority to choose future children with pre-implantation genetic diagnosis. Kennedy Institute of Ethics Journal.

[CR52] Tucker Bonnie Poitras (1998). Deaf culture, cochlear implants, and elective disability. The Hastings Center Report.

[CR53] Uppal Sharanjit (2006). Impact of the timing, type and severity of disability on the subjective well-being of individuals with disabilities. Social Science and Medicine.

[CR54] Vehmas Simo, Shakespeare Tom (2014). Disability, harm, and the origins of limited opportunities. Cambridge Quarterly of Healthcare Ethics.

[CR55] Wasserman David, Asch Adrienne (2012). Selecting for disability: Acceptable lives, acceptable reasons. The American Journal of Bioethics.

[CR56] Xiong W, Wang D, Gao Y, Gao Y, Wang H, Guan J, Lan L (2015). Reproductive management through integration of PGD and MPS-based noninvasive prenatal screening/diagnosis for a family with GJB2-associated hearing impairment. Science China Life Sciences.

[CR57] Yazdi AK, Davoudi-Dehaghani E, Anari MR, Fouladi P, Ebrahimi E, Sabeghi S, Eftekharian A (2018). The first successful application of preimplantation genetic diagnosis for hearing loss in Iran. Cellular and Molecular Biology (Noisy-le-Grand, France).

[CR58] Young Thomas (2001). Overconsumption and procreation: Are they morally equivalent?. Journal of Applied Philosophy.

